# Dr. Henry L. Day

**Published:** 1905-10

**Authors:** 


### OBITUARY.

---

DR. HENRY L. DAY, of Eau Claire, Wis., a graduate of the Medical Department, University of Buffalo, 1860, died of pleurisy at his home September 16, 1905, aged 66 years. Dr. Day formerly lived at Arcade, N. Y., and served as assistant surgeon of the 78th N. Y. Infantry during the Civil War. He was prominent as a physician at Eau Claire for 24 years, serving two terms as mayor of the city.

